# Prediction Efficiency of Postoperative Acute Kidney Injury in Acute Stanford Type A Aortic Dissection Patients with Renal Resistive Index and Semiquantitative Color Doppler

**DOI:** 10.1155/2019/4381052

**Published:** 2019-12-03

**Authors:** Huai Qin, Yaqiong Li, Nan Zhang, Tiezhu Wang, Zhanming Fan

**Affiliations:** ^1^Department of Ultrasound, Beijing Anzhen Hospital, Capital Medical University, Beijing Institute of Heart Lung and Blood Vessel Diseases, Beijing, China; ^2^Department of Cardiovascular Intensive Care, Beijing Anzhen Hospital, Capital Medical University, Beijing Institute of Heart Lung and Blood Vessel Diseases, Beijing Aortic Disease Center, Beijing, China; ^3^Department of Radiology, Beijing Anzhen Hospital, Capital Medical University, Beijing Institute of Heart Lung and Blood Vessel Diseases, Beijing, China; ^4^Department of Ultrasound, Hospital of Renmin University of China, Beijing, China

## Abstract

**Objectives:**

This study is aimed to evaluate the efficiency in early prediction of postoperative persistent acute kidney injury (PAKI) after surgery in acute Stanford type A aortic dissection (AAAD) patients by using Doppler renal resistive index (RRI) and semiquantitative color (SQC) Doppler grade, respectively.

**Methods:**

84 AAAD patients received Sun's surgical management, and 67 patients were enrolled. RRI and SQC Doppler grade were evaluated by ultrasonography, respectively, at 6 hours after surgery. Serum creatinine (sCr) was recorded before operation and at 24 hours, 48 hours, and 72 hours after operation. AKI grade was evaluated according to the classifications of the Acute Kidney Injury Network (AKIN). PAKI is defined as persistent oliguria and/or sCr elevation after 3 days. RRI and SQC Doppler grade were compared, respectively, between the PAKI and non-PAKI groups. Potential predictors were first tested by univariate logistic regression analysis, and a multivariate model was identified to determine the independent predictive ability of RRI and SQC Doppler grade for the PAKI. Receiver operating characteristic (ROC) analysis was performed to compare the diagnostic accuracy between RRI and SQC Doppler grade in early prediction of PAKI by using AKIN classifications as the reference standard.

**Results:**

Of a total of 67 patients enrolled during the study period, 21 (31.3%) patients suffered from PAKI and 8 (11.9%) patients required dialysis. There are significant differences in RRI (0.80 ± 0.09 vs. 0.70 ± 0.05, *P*=0.002) and SQC Doppler grade (*x*^2^=12.193, *P*=0.007) between the 2 groups with and without PAKI. Univariate analysis showed that RRI, SQC Doppler grade, length of stay in ICU, time of CPB, and length of stay in hospital were significant predictors of PAKI. RRI and the SQC Doppler grade remained independent predictors of PAKI. Area under the curve (AUC) of RRI was 0.855 (95% CI, 0.74–0.96) with cutoff value 0.725 (sensitivity 90.9% and specificity 71.1%), AUC of SQC Doppler grade was 0.642 (95% CI, 0.49–0.79) with cutoff value grade 2 (sensitivity 50% and specificity 73.3%).

**Conclusion:**

Both postoperative RRI and SQC Doppler grade are independent predictors for PAKI after surgery in AAAD patients. Both postoperative RRI and SQC Doppler grade can be obtained rapidly by bedside ultrasound, which is a good tool for early prediction for postoperative PAKI.

## 1. Introduction

The gold standard for the diagnosis of postoperative AKI is serum creatinine (sCr), but it is not used for early detection of AKI [1, 2]. Recent studies provide early diagnosis of AKI with several urine and blood markers [3–6]. Commonly used urine markers included neutrophil gelatinase-associated lipocalin (NGAL), cystatin C, kidney injury molecule 1 (KIM-1), interleukin-18 (IL-18), liver-type fatty acid-binding protein (L-FABP), and other more. Blood markers include cystatin C, uric acid, and monocyte chemoattractant protein-1 (MCP-1). Although these indicators have certain efficacy while intraoperative monitoring, urine and blood markers cannot be used to predict severe AKI [7].

Postoperative AKI is an ischemia-reperfusion injury, so renal perfusion is associated with AKI [8]. There are several imaging methods to evaluate renal perfusion. Computed tomography angiography (CTA) can evaluate renal perfusion, but the postcontrast AKI carries a risk of more permanent renal insufficiency [9, 10], which is also not suitable for daily bedside operation. Isotope renogram is a very sensitive, simple, and noninvasive method for evaluating renal parenchymal function [11], but it requires the patient to go to the fixed examination room and is also not suitable for the critically ill patients in the postoperative care unit.

Ultrasound is being used increasingly, especially in the intensive care unit (ICU) after operation, which has been recognized by the ICU doctors. The RRI measured by Doppler can be worked as a marker to predict AKI in some kinds of cardiac operations [12–15]. However, some other literatures do not consider RRI to be a good indicator of renal perfusion [16, 17]. RRI as a marker to predict AKI needs to be confirmed in more research studies. Moreover, intrarenal arcuate or interlobar arteries cannot be displayed when critical AKI occurred, RRI was hardly to be obtained, and the clinical application was limited. The color Doppler can intuitively and instantly observe renal perfusion, and semiquantitative evaluation can be acquired rather than an accurately quantitative renal perfusion assessment [18]. But few studies about this indicator are reported. The purpose of this study was to evaluate efficiency of RRI and SQC Doppler for prediction of PAKI after surgery in AAAD patients.

## 2. Patients and Methods

This study was conducted in accordance with the Declaration of Helsinki. This study was approved by the Ethics Committees of Beijing Anzhen Hospital, Capital Medical University. The committee agreed with using the data obtained from the postoperative patients in the ICU.

### 2.1. Patients

**Figure 1:**
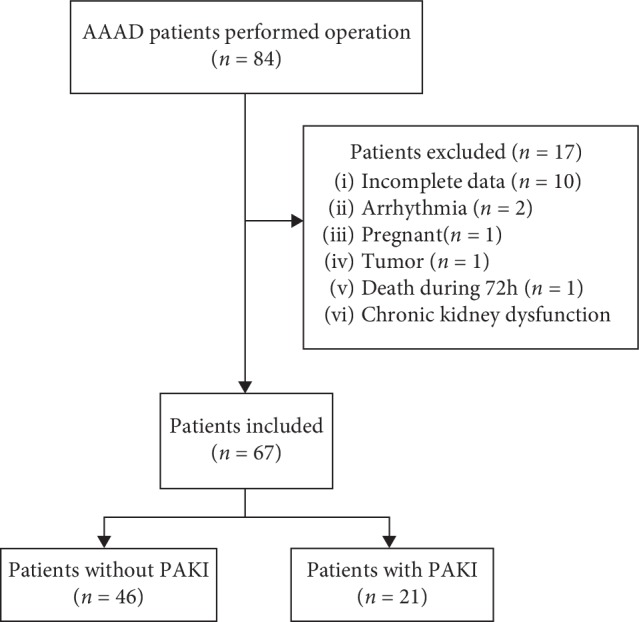
Flowchart of the study (AAAD: acute Stanford type A aortic dissection; PAKI: persistent acute kidney injury).

A prospective, observational study was performed during a nonconsecutive period (September 2017–March 2018) at Beijing Anzhen Hospital, cardiac surgery intensive care unit. Inclusion criteria were as follows: the AAAD patients received Sun's surgical operation with age >18 years and inclusion within 24 hours after ICU admission. Exclusion criteria were as follows: (1) history of chronic kidney dysfunction and preoperative renal injury; (2) pregnancy; (3) death during 72 hours after operation; (4) kidney tumor (*n*=1); (5) known renal artery stenosis; (6) arrhythmia; and (7) incomplete data. 84 AAAD patients received Sun's surgical operation, and 67 patients were analyzed (Figure 1), including 53 males and 14 females.

### 2.2. SQC Doppler Grade Evaluation

**Figure 2:**
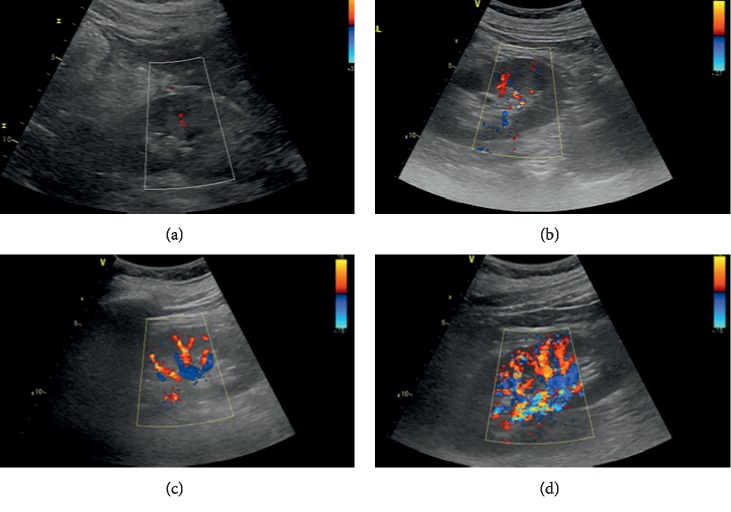
Semiquantitative color Doppler (SQC) grade. (a) Unidentifiable vessels were seen in the kidney; SQC grade was 0. (b) Vessels were seen in the renal hilum, but no vessels were seen in the parenchyma; SQC grade was 1. (c) Interlobar vessels were seen in most of the renal parenchyma; SQC grade was 2. (d) Arcuate arteries were seen in the entire field of view; SQC grade was 3.

All ultrasound measurements and evaluations were performed using a 3–5 MHz pulsed wave Doppler probes with GE equipment (Vivid E9, GE Healthcare, Horton, Norway) by one of the investigators. The kidneys were displayed along a longitudinal section with two-dimensional ultrasound at the renal hilum slice. The color Doppler gain was set to minimal background noise. Each renal perfusion was evaluated by color Doppler using the semiquantitative scale (Table 1 and Figure 2) [19].

As the perfusion in the bilateral kidney is diverse in AAAD patients, the renal perfusion grade was defined as the average.

### 2.3. RRI Measurement

Renal blood flow was located along a longitudinal section showing the renal hilum [18] and interlobar arteries. Sampling any side midinterlobar artery (IRA) with an angle of blood stream <30°. Set to minimal background noise, the Doppler gain can obtain at least three similar continuous waveforms. In each kidney, interlobar artery peak systolic velocity and end diastolic velocity were measured three times at the Doppler spectrum. RRI was calculated by the equation as follows:

(1) $$\begin{array}{c} \text{RRI}=\frac{\left(\text{peak systolic velocity}-\text{end diastolic velocity}\right)}{\text{peak systolic velocity}}. \end{array}$$

Because of diversity at the bilateral kidneys perfusion in AAAD patients, RRI was recorded as average.

### 2.4. Study Design

Ultrasound SQC Doppler grade and RRI measurements were performed at 6 hours after surgery.

We started to measure the RRI and SQC Doppler grade of 14 kidneys in 7 patients by a double-blind method, in order to compare the consistency of the two operators.

sCr was recorded before operation (creatinine was defined as the last known creatinine value before operation) and 24 hours, 48 hours, and 72 hours after operation. AKI was classified into three grades according to the AKIN classification system [20]. PAKI was defined as persistent oliguria or serum creatinine elevation within at least 3 days [21].

### 2.5. Statistical Analysis

Quantitative and qualitative data are expressed as median ± standard deviation and numbers (percentages), respectively. The nonparametric test was used for categorical variables, and the *t*-test was for continuous variables between groups with and without PAKI. Counting data of patient outcome and SQC Doppler grade were analyzed by the *x*^2^ test. Difference of RRI and SQC Doppler grade between the two operators was compared by using the Kendall test. Potential predictors were first tested by univariate logistic regression analysis, to determine the predictive ability of the RRI and SQC Doppler grade for the development of PAKI. A multivariate model was identified applying a p-entry and removed the value of *p* less than 0.05.

Using AKIN as the standard, receiver operating characteristic (ROC) analysis was performed on RRI and SQC Doppler grade to assess the efficiency of the two markers. All tests were two sided, and a probability value of less than 0.05 was considered statistically significant. SPSS 20.0 (SPSS Inc., IBM, USA) was used to analyze the data.

## 3. Results

### 3.1. Operator Consistence

The Kendall coefficient of RRI and SQC Doppler grade between the two operators was 0.827 (*n*=14, *P* *≦* 0.001) and 0.693 (*n*=14, *P*=0.027).

#### 3.1.1. Primary Outcomes

Of a total of 67 patients during the study period, 21 (31.3%) patients suffered from PAKI and 8 (11.9%) patients required dialysis. Base characteristics were similar between the two groups (PAKI and without PAKI) except for preoperative sCr (Table 2). Intraoperative risk factors and postoperative influencing factors were similar between the two groups (PAKI and without PAKI) except for time of CPB and CRRT, length of stay in hospital, and length of stay in ICU (Table 3).

#### 3.1.2. Outcomes of RRI and SQC Doppler Grade

**Figure 3:**
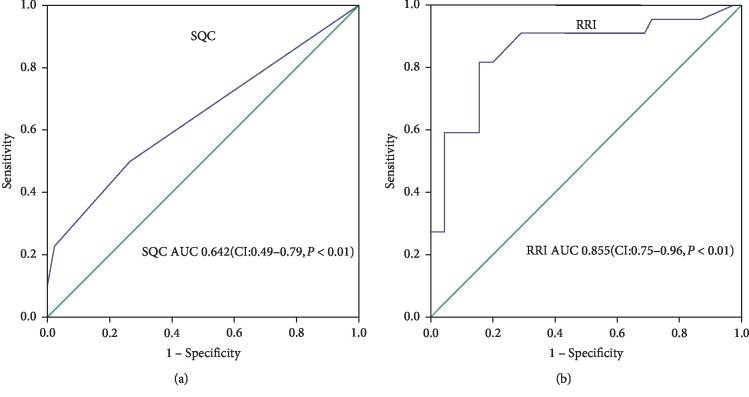
Prediction of postoperative persistent acute renal injury with ROC curves using postoperative RRI (a) and SQC Doppler grade (b).

1. There is a significant difference in postoperative RRI in groups PAKI and without PAKI (0.79 ± 0.09 vs. 0.72 ± 0.06, *P*=0.002). There is also a significant difference in the SQC Doppler grade in between the two groups (*x*^2^=12.193, *P*=0.007) (Table 4).
2. Since 0 appears in the data of patients without PAKI, we combine SQC Doppler grade 0 and 1 to SQC Doppler grade 1 for univariate analysis. It shows that both RRI and SQC Doppler grade 2 were significant predictors of PAKI; besides, the other three significant predictors were length of stay in ICU, time of CPB, and length of stay in hospital (Table 5).
3. Similar to univariate regression analysis, we combine SQC Doppler grade 0 and 1 to SQC Doppler grade 1 for multivariate regression analysis. To determine whether RRI and SQC Doppler grade 2 were independent predictors of PAKI, the other three most significant variables in univariate analysis as confounders were analyzed for PAKI prediction in multivariate analysis, in addition to RRI and SQC Doppler grade. RRI remained a significant predictor of PAKI, independent of length of stay in ICU, time of CPB, and length of stay in hospital. Similarly, the SQC Doppler grade is a predictor of PAKI independent of length of stay in ICU, time of CPB, and length of stay in hospital (Table 6).
4. AUC of postoperative RRI to predict PAKI was 0.855 with cutoff value 0.725 (95% CI, 0.74–0.96) (sensitivity 90.9% and specificity 71.1%) (Figure 3(a)). AUC of the SQC Doppler grade to predict PAKI was 0.642 (95% CI, 0.49–0.79) with cutoff value grade 2 (sensitivity 50% and specificity 73.3%) (Figure 3(b)).

## 4. Discussion

This prospective study shows that both postoperative RRI and SQC Doppler grade are significant predictors of postoperative persistent AKI after AAAD surgery.

The two operators have good consistence at the RRI calculation and SQC Doppler grade evaluation, and the Kendall coefficient was 0.827 (*n*=14, *P* *≦* 0.001) and 0.693 (*n*=14, *P*=0.027), respectively. Close to the previous study [19], the technology with good reproducibility between the 2 operators avoided technique bias. The accuracy of RRI calculation and SQC Doppler grade evaluation still needs further study in larger scale samples and multiple centers.

The incidence of postoperative PAKI in our study was 31.3%, and 11.9% patients required dialysis, which agrees with the previous study [22].

In this study, postoperative RRI was higher in PAKI patients than that in non-PAKI patients. Univariate and multivariate regression analysis certified that postoperative RRI was an independent predictor for developing of postoperative PAKI. Although preoperative sCr was different between groups with and without PAKI, but by the univariate regression analysis preoperative sCr is not a significant predictor, therefore not included in the multivariate analysis. The result of the AUC of postoperative RRI was 0.855 for predicting PAKI with a cutoff value of 0.725 (95% CI, 0.49–0.79) (sensitivity 90.9%, specificity 73.3%). The cutoff value 0.725 was the same as that of our previous research [23], which indicates that RRI predicts postoperative AKI and has a fairly stable value in AAAD patients in our center. But it was higher than some studies [24, 25] and lower than other literatures [26, 27]. We selected the average RRI in bilateral kidneys because of each kidney perhaps causing by different perfusion from the true or false lumen in AAAD patients. Furthermore, RRI cannot be evaluated in one of the bilateral kidneys because of poor kidney perfusion in 3 patients. It can result in deviations in the RRI values. Although multiple pathogenic pathways existed between RRI and AKI development [24, 28, 29], in conclusion our study certificated that postoperative RRI with cutoff value 0.725 was an independent predictor for the development of postoperative PAKI in AAAD patients.

In this study, patients below SQC Doppler grade 2 accounted for 71.4% in the PAKI group and 45.7% in the non-PAKI group. Univariate regression analysis certificated that SQC Doppler grade 2 was a significant predictor for postoperative PAKI; our study is the first to certify that SQC Doppler grade was an independent predictor of postoperative PAKI by multivariate regression analysis. In this study, when we set the SQC Doppler grade less than 2 as a predictor for persistent AKI, AUC of SQC Doppler grade to predict PAKI was 0.642 (95% CI, 0.49–0.79) with sensitivity 50% and specificity 73.3%. The precision of SQC Doppler grade in predicting postoperative PAKI was poorer than postoperative RRI, which is in accordance with the recent study [30]. In critically ill patients, persistent AKI is considered to be the result of acute tubular necrosis which is caused by initially reduced renal perfusion [31]. The finding can help solving the problem of rapid, flexible, inexpensive, and noninvasive in patients after surgery at the intensive care unit.

In conclusion, both postoperative RRI and SQC Doppler grade are independent predictors for PAKI after surgery in AAAD patients. Moreover, both postoperative RRI and SQC Doppler grade can be obtained rapidly by bedside ultrasound, which is an important diagnostic tool in the case of clinical conditions that might impair kidney function [32].

There was no significant difference in demographic and clinical information between the PAKI and without PAKI groups except for preoperative sCr. However, preoperative sCr was not an independent predictor of postoperative AKI by univariate and multivariate analysis in this research. Moreover, to avoid intrarenal hemodynamic changes causing bias, we measured RRI and evaluated the kidney perfusion by ultrasound in the period with mechanical ventilation, supine, during a narrow range of PaCO_2_. Because the level of breathing, body position and PaCO_2_ may affect RRI measurement. [33].

We have two strengths: (1) our study is the first to certify SQC Doppler grade as an independent predictor of postoperative PAKI by multivariate regression analysis; (2) the cutoff value 0.725 of postoperative RRI was the same as that of our previous research, which indicates it will have good applicability in AAAD patients in our center.

There are some limitations in our study. (1) The blood flow in the kidney was not easily observed in some obese patients or severe AKI patients. Bias in judgment of the results may exist. (2) The length of the six months study period and the limitation of the small sample, so the statistical results have a large confidence interval. (3) This was a single-center and single-disease study. A further study with wider range of applications, larger sample, and multiple centers is required.

## 5. Conclusion

Both postoperative RRI and SQC Doppler grade are independent predictors for PAKI after surgery in AAAD patients.

Both postoperative RRI and SQC Doppler grade can be obtained rapidly by bedside ultrasound, which is a good tool for early prediction for postoperative PAKI.

## Figures and Tables

**Table 1:** Semiquantitative color Doppler scale in renal perfusion.

Grade 0	Unidentifiable vessels
Grade 1	Few vessels visible in the vicinity of the hilum
Grade 2	Hilar and interlobar vessels visible in most of the renal parenchyma
Grade 3	Renal vessels identifiable until the arcuate arteries in the entire field of view

**Table 2:** Base characteristics between groups with PAKI and without PAKI.

	All subjects with operation (*n*=67)	Subjects without PAKI (*n*=46)	Subjects with PAKI (*n*=21)	*P* value
Male (%)	53 (79.1)	38 (82.6)	15 (71.4)	0.340
Age in years	46.48 ± 10.64	45.61 ± 10.76	48.38 ± 10.38	0.360
BMI (kg/m^2^)	27.85 ± 6.35	28.04 ± 7.18	27.45 ± 4.11	0.729
Hypertension (%)	58 (86.5)	39 (84.7)	19 (90.4)	0.709
Diabetes (%)	2 (2.9)	1 (2.0)	1 (4.7)	1.000
Smoking (%)	58 (86.5)	38 (82.6)	20 (95.2)	0.187
Drinking (%)	12 (17.9)	6 (13.0)	6 (28.5)	1.000
Preoperative sCr (*μ*mol/L)	88.1 ± 50.9	78.5 ± 26.6	109.2 ± 79.3	0.021
MAP (mm·Hg)	83.3 ± 12.4	83.8 ± 12.4	83.5 ± 12.5	0.915
HR (beats min^−1^)	96 ± 17	97 ± 17	96 ± 15	0.950
CVP (mm·Hg)	9.4 ± 3.0	9.0 ± 2.0	10.1 ± 4.5	0.155
PaCO_2_ (mm·Hg)	46.9 ± 8.5	46.5 ± 7.6	47.8 ± 10.4	0.559
PaO_2_ (mm·Hg)	158.8 ± 274.0	133.6 ± 57.3	213.9 ± 448.3	0.269
EF (%)	58.4 ± 7.2	59.4 ± 6.6	56.2 ± 8.0	0.086

BMI: body mass index; MAP: mean arterial pressure; HR: heart rate; CVP: central venous pressure; EF: ejection fraction.

**Table 3:** Intraoperative and postoperative characteristics between groups with PAKI and without PAKI.

		All subjects (*n*=67)	Subjects without PAKI (*n*=46)	Subjects with PAKI (*n*=21)	*P* value
	Time of CPB (min)	205 ± 43	198 ± 43	221 ± 40	0.037
	Time of aortic cross clamping (min)	123 ± 33	125 ± 160	120 ± 23	0.059
	Time of DHCA (min)	25.0 ± 11.3	23.3 ± 8.8	28.9 ± 14.8	0.889
	Urine output (ml)	1848 ± 1065	1910 ± 958	1714 ± 1285	0.489

*Intraoperative drugs*					
	Dopamine	3.57 ± 2.55	3.48 ± 2.50	3.76 ± 2.60	0.676
	Adrenaline	0.025 ± 0.020	0.024 ± 0.021	0.026 ± 0.018	0.802

*Outcomes*					
	Postoperative sCr (*μ*mol/L)	140.32 ± 129.62	116.72 ± 138.56	188.61 ± 94.55	
	CRRT (%)	8 (11.9)	2 (4.3)	6 (28.6)	0.009
	Length of stay in hospital (days)	14.5 ± 8.4	13.4 ± 1.8	16.8 ± 9.4	0.003
	Length of stay in ICU (days)	4.7 ± 3.7	3.8 ± 3.5	6.6 ± 3.5	0.009

CPB: cardiopulmonary bypass; DHCA: deep hypothermic circulatory arrest; CRRT: continuous renal replacement therapy.

**Table 4:** Comparison of RRI and semiquantitative color Doppler grade between groups with PAKI and without PAKI.

	All subjects (*n*=67)	Subjects without PAKI (*n*=46)	Subjects with PAKI (*n*=21)	*P* value
*RRI*				
	0.75 ± 0.08	0.72 ± 0.06	0.79 ± 0.09	0.002

*Semiquantitative color Doppler grade*				
0	3 (4.4%)	0 (0%)	3 (14.3%)	
1	6 (8.9%)	1 (2.2%)	5 (23.8%)	
2	27 (40.3%)	20 (29.8%)	7 (33.3%)	
3	31 (46.2%)	25 (54.3%)	6 (28.6%)	
				0.007

**Table 5:** Univariate regression analysis for PAKI.

	OR	OR (95% CI)	*P*-value
Male	0.377	0.114–1.245	0.109
Age	1.025	0.976–1.078	0.322
BMI	0.997	0.919–1.082	0.944
Hypertension	1.842	0.350–9.709	0.471
Preoperative sCr (*μ*mol/L)	1.106	0.999–1.033	0.063
MAP (mm·Hg)	0.957	0.956–1.039	0.869
HR (beats min^−1^)	0.995	0.964–1.026	0.747
CVP (mm·Hg)	1.138	0.941–1.377	0.182
PaCO_2_ (mm·Hg)	1.018	0.951–1.081	0.562
PaO_2_ (mm·Hg)	1.001	0.999–1.103	0.377
EF (%)	0.958	0.878–1.045	0.330
Time of CPB (min)	1.015	1.002–1.028	0.021
Time of aortic cross clamping (min)	1.000	0.996–1.004	0.972
Time of DHCA (min)	1.037	0.991–1.085	0.112
Dopamine	1.061	0.865–1.301	0.571
Adrenaline	2.241	0.001–1.755	0.950
Urine output (ml)	1.000	0.999–1.000	0.439
CRRT	0.049	0.006–0.429	0.006
Length of stay in hospital (days)	1.085	1.010–1.166	0.025
Length of stay in ICU (days)	1.670	1.256–2.221	<0.001
RRI	6.553	2.454–17.503	<0.001
SQC Doppler grade			
2	27.429	2.912–258.387	0.004
1	1.200	0.360–4.000	0.767

**Table 6:** Multivariate regression analysis for PAKI.

	OR	OR (95% CI)	*P* value	OR	OR (95% CI)	*P* value
Time of CPB (min)	1.006	0.989–1.023	0.515	1.004	0.991–1.023	0.414
Length of stay in hospital (days)	0.962	0.861–1.073	0.486	0.957	0.848–1.079	0.471
Length of stay in ICU (days)	1.512	1.075–2.218	0.018	1.733	1.204–2.493	0.003
RRI	4.110	1.396–12.013	0.010			
SQC Doppler grade 2				19.380	1.406–267.208	0.027
1				1.798	0.395–8.197	0.448

## Data Availability

The data used to support the findings of this study are available from the corresponding author upon request.
